# Comparison of DNA extraction procedures for detection of *Mycoplasma bovis* directly from extended bovine semen straw samples using a commercial *M. bovis* PCR

**DOI:** 10.1186/s12917-024-04333-z

**Published:** 2024-10-26

**Authors:** Emma Taylor, Alannah Deeney, Colin Birch, Georgia Mayne, Anne Ridley

**Affiliations:** 1 Mycoplasma Team, Department of Bacteriology, Animal and Plant Health Agency, Woodham Lane, New Haw, Addlestone, Surrey KT15 3NB UK; 2 Department of Epidemiological Sciences, WOAH Collaborating Centre for Risk Analysis and Modelling, Animal and Plant Health Agency, Woodham Lane, New Haw, Addlestone, Surrey KT15 3NB UK

**Keywords:** Mollicutes, Mycoplasma, *Mycoplasma bovis*, Semen, Polymerase chain reaction, Real-time PCR, Validation

## Abstract

**Background:**

*Mycoplasma bovis* is a global pathogen of cattle but was detected for the first time in New Zealand in 2017, triggering a response under their Biosecurity Act as an “unwanted organism”. Following a lengthy eradication campaign, the Ministry of Primary Industries (MPI) now requires all bovine semen destined for export to New Zealand to be screened with an *M. bovis*-specific real-time PCR (rtPCR) compliant with amended import health standard (IHS) test requirements aimed at preventing the accidental importation of *M. bovis.* The standard stipulates that semen samples cannot be centrifuged prior to DNA extraction. To comply with these strict requirements, one of the listed tests was validated together with different DNA preparation steps and compared with existing in-house procedures. DNA was extracted from semen straws using the current in-house semi-automated platform procedures for processing culture, tissue and body fluid sample submissions and was compared with the stipulated test requirements. DNA from centrifuged and unspun semen samples spiked with *M. bovis* was also compared.

**Results:**

The rtPCR had a sensitivity and specificity of 100% (95% confidence interval = 79–100% and 74–100%, respectively) when testing DNA from other *Mycoplasma* species or bovine semen spiked with the latter, with a high level of repeatability for within- and between- run replicates. The consistent limit of detection was 0.001 pg/µl *M. bovis* DNA and between 5.3 × 10^2^ and 7.5 × 10^2^ CFU/ml *M. bovis* when artificially spiked in semen. DNA extracted using the KingFisher Flex was detected with lower Cq values than the Maxwell 16, but the comparable improvements in sensitivity were mainly associated with non-centrifuged samples (*p* < 0.001). None of the procedures tested impeded the detection sensitivity of *M. bovis* in the presence of competitor organisms *Acholeplasma laidlawii*, *Mycoplasma bovigenitalium* and *Ureaplasma diversum*, confirming *M. bovis* specificity of the *polC* target.

**Conclusions:**

Under the experimental conditions applied, this rtPCR test efficiently detected *M. bovis* in extended bovine semen straw samples from DNA extracted using both semi-automated extraction platforms, irrespective of prior centrifugation of extended semen.

**Supplementary Information:**

The online version contains supplementary material available at 10.1186/s12917-024-04333-z.

## Background

*Mycoplasma bovis* is an important cattle pathogen, belonging to the class Mollicutes. It was first identified in the USA in 1961 from a severe case of bovine mastitis [1] but is now found worldwide [2], causing calf pneumonia [3], arthritis [4], mastitis [5], otitis media [6] and other conditions in cattle. *M. bovis* causes substantial economic losses, with an impact on welfare in the cattle industry [2, 7]. Although animal movement [8] and close contact with infected animals are the predominant routes of transmission, infection risks also include airborne transmission [9], ingestion of contaminated colostrum or milk [6], contaminated equipment and environment [10, 11]. Artificial insemination and embryo transfer have more recently been implicated as possible sources of infection [12–14].

In 2017, *M. bovis* was first detected in New Zealand and major efforts are still ongoing, led by its Ministry of Primary Industries (MPI), to eradicate this pathogen from all New Zealand herds [15], although eradication is close to completion. It is believed *M. bovis* was first introduced into the country in July 2015 [16]. Key pathways for the introduction of *M. bovis*, such as imported cattle or other live animals, bovine embryos, bovine semen, feed and used farm equipment have been investigated as potential sources of the outbreak [17]. Although the source of the outbreak has yet to be proven [18, 19], an outbreak of *M. bovis* in Finland in 2013 was attributed to the importation of *M. bovis*-infected semen [13]. The absence of live cattle imports to New Zealand since 2013 [17], combined with retrospective PCR testing [19] strongly suggests that infected imported semen is a plausible source of the New Zealand outbreak.

The New Zealand Animal IHS and associated Diagnostic Tests, Vaccines, Treatments and Laboratories for Post-Arrival Testing for Animal Import Health Standards have subsequently been revised to include a requirement to screen all semen batches destined for export to New Zealand using an *M. bovis*-specific PCR to minimise the chances of importing infected semen into the country [20].

Within the Mycoplasma Team at the Animal and Plant Health Agency (APHA), which is based in the United Kingdom, the standard approach for identification of a variety of mollicutes, including *M. bovis*, is to employ culture and endpoint PCR-denaturing gradient gel electrophoresis (DGGE). This method is applied to diagnostic sample types including lung and mammary tissue, respiratory lavage, joint aspirate, milk, blood, swab samples from various tissue surfaces and less commonly, semen and cerebrospinal tissues [21]. A conventional specific PCR is also used to confirm *M. bovis* where required [22]. A number of other conventional PCRs [23–32] have been used, predominantly on milk and a variety of respiratory sample types. Only a few PCRs have previously been reported for testing semen samples [13, 32–34].

The updated IHS states two semen straws per batch of extended semen are to be tested with one of three specified rtPCRs (VetMAX™ *M. bovis* PCR kit [ThermoFisher Scientific]; KaspRT PCR [35] and a rtPCR designed by Rossetti et al. [29]). A previous European inter-laboratory comparison of the commercial VetMAX™ *M. bovis* PCR kit (ThermoFisher Scientific) involving APHA [22], showed a higher sensitivity compared to conventional PCR methods and appeared robust and easy to use in our hands when assessed with bronchoalveolar fluid samples.

The IHS has also stated requirements regarding preparation of DNA template from semen for *M. bovis* PCR screening; these include preparation using the manual Qiagen DNA-Mini Kit (Qiagen), or automated KingFisher Sample Purification Systems (ThermoFisher Scientific), using the MagMAX™ CORE Nucleic Acid Purification Kit (ThermoFisher Scientific), or the MagMAX Total Nucleic Acid Isolation Kit (ThermoFisher Scientific), according to manufacturers’ instructions for semen [36]. Additional stipulations included: DNA elution volumes must be 90–100 µl and semen processed should not be centrifuged, nor should pellets be washed with PBS when using the Qiagen DNA-Mini Kit [36]. Culture PCR-DGGE procedures at APHA [21] involve overnight incubation of semen samples in *Mycoplasma* liquid culture media at 37 °C (+/- 2 °C) in an atmosphere of 5% CO_2_, followed by centrifugation, suspension of the pellet in lysis buffer and a heat lysis step prior to extraction using the automated Maxwell 16 Instrument (Promega).

Therefore, to comply with IHS specifications and to enable testing of bovine semen derived from Great Britain in support of international trade globally, including New Zealand, the VetMAX™ *M. bovis* PCR kit was chosen for validation based on the aforementioned previous experience with different sample types. The validation included a performance comparison with different DNA extraction protocols based on both the semi-automated Maxwell 16 and the KingFisher Flex platforms in place at APHA. Centrifuged (our normal procedure) and unspun samples (stipulated by IHS) were also compared on both DNA extraction platforms to compare efficiency of extraction of *M. bovis* DNA from extended bovine semen straws without centrifugation or washing of pellets, irrespective of automated extraction protocols used.

## Methods

### Bacterial isolates

Samples of DNA used for preliminary sensitivity and specificity studies were from control strains of *Mycoplasma* spp. currently in use as controls for PCR-DGGE and, as appropriate, endpoint specific PCR tests. *M. bovis* has not been recovered from UK semen samples to date. Additionally, the *M. bovis* strains 353B08 and 2B19, used for the limit of detection studies, were recovered from field respiratory disease cases and selected to represent genomically divergent strains circulating in Great Britain that are present globally, while *M. bovigenitalium* strains NCTC 10122 and 129B17 represented type and a field strain from cattle lung, respectively; *Acholeoplasma laidlawii* 158B98 and *Ureaplasma diversum* strain 382B16 were also isolated from cattle respiratory samples as samples from urogenital origin were not available; all had been confirmed by PCR-DGGE, with additional sequencing of the PCR product.

### Semen straws

Extended bovine semen straws (*n* = 524) from each of three bulls 1 (*n* = 173), 2 (*n* = 173) and 3 (*n* = 178) were kindly provided by a commercial bovine breeding company (Cogent Breeding, Tattenhall, UK). Representatives from individual batches of straws were pre-screened prior to the start of this work to ensure negativity for *M. bovis* via the VetMAX™ *M. bovis* rtPCR and a Mollicutes PCR-DGGE to confirm their negativity (data not shown). DNA extraction was performed using the Maxwell 16 automated extraction platform (Promega UK, Southampton, UK) as described below.

### Processing semen straws

Semen was removed from straws by piercing the straw ends with a soldering iron with semen released into either 1.5 ml Eppendorf tubes (for testing individual samples) or into 15 ml conical Falcon^®^ centrifuge tubes (VWR International, Lutterworth, UK) for pooling.

### DNA extraction

For DNA extraction using the Maxwell 16 automated extraction platform (Promega UK, Southampton, UK), 200 µl semen samples were pre-digested with 20 µl proteinase K (Qiagen Ltd-UK, Manchester, UK) in 180 µl ATL lysis buffer (Qiagen) at 55 °C for 3 h, prior to DNA extraction using the Maxwell 16 Blood DNA Purification Kit (Promega) according to the manufacturer’s instructions. Centrifuged samples, were spun at 17,000 g for 3 min in a 17R Fisher Scientific Micro Centrifuge (Fisher Scientific, Loughborough, UK) and the pellet was resuspended in 1 ml phosphate-buffered saline (PBS), following removal of the supernatant. Samples were spun again at 17,000 g for 3 min and the supernatant removed before the pellets were resuspended in proteinase K and ATL lysis buffer for a pre-digestion step at 55 ^o^C, prior to DNA extraction as described above.

For DNA extraction using the KingFisher Flex (ThermoFisher Scientific, Paisley, UK), a modified protocol recommended by Thermofisher Technical Experts (Erwin van der Wal, Thermofisher, personal communication) was applied. Briefly, 200 µl of lysis buffer from the MagMAX™ CORE Nucleic Acid Purification Kit (ThermoFisher Scientific) was added to each sample, followed by 210 µl of diluted proteinase K (200 µl PBS + 10 µl proteinase K). Samples were then incubated at 70 °C for 30 min. Following incubation, 400 µl binding buffer and 20 µl magnetic beads were added to the samples before transferring to a 96-well deep plate (ThermoFisher Scientific), with DNA extraction thereafter carried out on the KingFisher Flex, according to manufacturer’s instructions, using the KF_MMC_no-heat script. Samples undergoing centrifugation were spun for 5 min at 3,000 g, but 100 µl of the supernatant was removed prior to the addition of lysis buffer to ensure even resuspension of the pellets.

### VetMAX™ *M. bovis* rtPCR and PCR-DGGE

The VetMAX™ *M. bovis* rtPCR (ThermoFisher Scientific) was performed according to manufacturer’s instructions, where 20 µl of the mastermix ‘3 - Mix MPBO’, was added to 5 µl of the sample DNA, the positive control (‘4a - EPC MPBO’) and the negative control (nuclease-free water). The PCR was run on the AriaMx Real-Time PCR system (Agilent Technologies, Cheshire, UK) according to manufacturer’s instructions, but with modifications to cycle cut-off (Cq) values. The kit specifies Cq values < 45 as positive for *M. bovis* but based on communication with MPI (Jonathon Foxwell, personal communication) we initially determined positivity as Cq < 40.0. However, as data accumulated this was subsequently modified with Cq values > 36.0 but < 40.0 considered inconclusive in the analysis. The FAM-NFQ *M. bovis polC* gene target was detected using the FAM optical cartridge, with the VIC-NFQ labelled internal PCR control (IPC), targeting bovine DNA, detected using the HEX optical cartridge.

PCR-DGGE was performed as previously described [21] to amplify the V3 region of the 16S rDNA from 1 µl of extracted DNA using a conventional PCR and DGGE to analyse the PCR amplicons using INGENY phorU 232 apparatus (GRI Molecular Biology, Essex, United Kingdom) and Thistle Scientific VS20WAVE-DGGE (Thistle Scientific Ltd, Rugby, UK) electrophoresis systems. Gels were stained with 1X SYBR™ Gold (ThermoFisher Scientific) and visualised under UV light using the gelLITE Gel Documentation System (Thistle Scientific Ltd, Warwickshire, UK) and GenePIX software (Version 1.8.2.0, Thistle Scientific Ltd).

### Endpoint *M. bovis*-specific PCR

One microlitre of DNA was added to 0.5 µl of AmpliTaq Gold™ polymerase (5 U/µl, Applied Biosystems, Warrington, UK), 1 µl of 25 µM primers MBOUVRC2-L (5′-TTACGCAAGAGAATGCTTCA-3′) and MBOUVRC2-R (5′-TAGGAAAGCACCCTATTGAT-3′) [23], 2 µl of 10 mM dNTPs, 5 µl 10X Gold Buffer (Applied Biosystems), 6 µl of 25 mM MgCl_2_ and 33.5 µl of nuclease-free water. The PCR cycling conditions were 95 °C for 5 min, and 33 cycles of 94 °C for 30 s, 60 °C for 30 s and 72 °C for 90 s, followed by 72 °C for 7 min. Twenty microliters of DNA were loaded onto a 2% ethidium bromide E-gel (Invitrogen) and run for 30 min on the E-Gel^®^ PowerBase™ System Version 4 (Invitrogen). Gels were visualised under UV light. A 1,626 bp band matching the control sample indicated positivity of *M. bovis*.

### Assessing analytical specificity of the VetMAX rtPCR

To test analytical specificity, the VetMAX rtPCR was screened against a panel of 17 DNA samples (diluted to 1 pg/µl) from Mollicutes and *Campylobacter fetus* spp. identified by the APHA from bovine hosts over the last two decades [21]. The panel was prepared from stored cultures from the APHA collection, which were DNA extracted using the Maxwell 16 and included: *Mycoplasma agalactiae*; *M. alkalescens*; *M. arginini*; *M. bovigenitalium*; *M. bovirhinis*; *M. bovoculi*; *M. californicum*; *M. canadense*; *M. canis*; *M. dispar*; *M. fermentans*; *M. mycoides* subsp. *mycoides; M. preputii* (now redesignated as *M. tauri sp.* Nov [37]), *M. verecundum* and *U. diversum*. *Campylobacter fetus* subsp. *fetus* and *Campylobacter fetus* subsp. *veneralis*, were also included due to their association with bovine genital campylobacteriosis, which can lead to impaired reproductive performance [38]. Samples were tested in duplicate on two separate occasions.

### Diagnostic specificity from archived diagnostic DNA samples

Owing to the paucity of samples from semen in our collection that had been identified as harbouring *M. bovis* or other mollicutes by PCR DGGE, the VetMAX rtPCR was used to screen DNA from a panel of DNA samples (*n* = 25) extracted from different field sample types submitted for diagnostic testing, with selections made according to the original PCR-DGGE result. DNA samples extracted using the Maxwell 16 [22] were either positive for *M. bovis* alone (*n* = 4); positive for *M. bovis* in the presence of other *Mycoplasma* or non-*Mycoplasma* mollicutes including *U. diversum* (*n* = 12); positive for *Mycoplasma* species other than *M. bovis* (*n* = 5) or were negative for any known *Mycoplasma* spp. or other mollicutes (*n* = 4). The VetMAX rtPCR was compared with freshly run PCR-DGGE and specific endpoint *M. bovis* PCR results.

### Analytical sensitivity

The limit of detection was determined by performing serial dilutions of purified DNA from *M. bovis* NCTC 10131 (from 100 pg/µl to 0.00001 pg/µl), where DNA was diluted 1:10 (4 µl DNA in 36 µl Elution Buffer [Promega]). The DNA concentration was determined using the Qubit 2.0 Fluorometer (ThermoFisher Scientific). Each dilution was tested by the VetMAX rtPCR in triplicate and the experiment was conducted twice. The limit of detection was determined as the lowest concentration, where the replicates across the two PCR runs were consistently positive for a given dilution. The dilutions were used to calculate the PCR efficiency, regression coefficient (R^2^) and slope using the Agilent Aria 1.5 software (Agilent Technologies).

The limit of detection in semen was determined by serial dilution of *M. bovis* strain 353B08 (1.6 × 10^9^ CFU/ml) from 10^7^ CFU/ml to 10^0^ CFU/ml, in pooled bovine semen straw samples, and screening with the VetMAX rtPCR. Total sample volumes of 200 µl were maintained throughout the experiment to represent the total volume of semen typically retrievable from a straw, which was pre-determined by measuring the volume of semen from ten semen straws using a pipette following release into 1.5 ml Eppendorf tubes. *M. bovis* 353B08 was stored in aliquots of predetermined concentration for use in all experiments, to ensure consistency between replicate experiments for assessing differences in efficiencies of DNA extraction procedures. In brief, 10 µl of 1.6 × 10^9^ CFU/ml *M. bovis* strain 353B08 was diluted to 10^7^ CFU/ml in 190 µl pooled bovine semen. A ten-fold serial dilution using 20 µl of 10^7^ CFU/ml *M. bovis* in 180 µl pooled semen was then performed from 10^7^ to 10^0^ CFU/ml. Samples were incubated at 37 °C (± 2 °C) for one hour to allow *M. bovis* to bind to the sperm [12]. Following incubation, two drops of each semen sample from a pastette were used to inoculate 3 ml of Eaton’s 0134 broth [39], which was incubated at 37 °C (+/- 2 °C) in an atmosphere of 5% CO_2_ for up to two weeks, which allowed demonstration that the spiked sample contained viable organisms. Two sets of serial dilutions prepared in duplicate were performed for each experiment to mimic the use of two sets of semen straws required by MPI for testing as they specify a minimum of two semen straws are required to be tested per animal to produce two test results per batch of straws. One set of two straws was centrifuged prior to DNA extraction on the Maxwell 16 or KingFisher Flex, while the other pair was not centrifuged. Each experiment was performed in triplicate, on separate days, resulting in six replicates per condition.

All the results from the spiking experiments were converted to CFU/ml to identify the limit of detection, using standard curves generated in the Agilent Aria 1.5 software (Agilent Technologies) from colony counts of serial dilutions of bacterial cultures of *M. bovis* strain 353B08 plated in triplicate on Eaton’s agar for each experiment. Standard curves were generated for samples processed by the KingFisher Flex and Maxwell 16 separately. The DNA from each dilution was also tested with PCR-DGGE and the endpoint *M. bovis*-specific PCR to compare the limit of detection with the VetMAX rtPCR.

### Detection of *M. bovis* in supernatants from centrifuged samples

Supernatants from two sets of centrifuged samples from semen spiking experiments on the Maxwell 16 and KingFisher Flex were processed according to the protocol of the respective DNA extraction method outlined above and screened with the VetMAX rtPCR to detect *M. bovis* DNA. The standard deviation was calculated between the Cqs of the supernatants and the respective centrifuged semen samples to show the difference in Cqs and results were converted to CFU/ml as described above.

### Analytical specificity in semen

Following assessment of DNA extraction methods, analytical specificity of the extraction and VetMAX rtPCR protocol was then tested by screening spiked pooled straws using the VetMAX rtPCR. Isolates for spiking reflected species known to infect the urogenital tract of bovines and isolated from semen (*M. bovigenitalium* [NCTC 10122], *A. laidlawii* [158B98], and *U. diversum* [382B16]). Ten microlitres of *A. laidlawii* (4.3 × 10^8^ CFU/ml), *M. bovigenitalium* (1.1 × 10^8^ CFU/ml) and *U. diversum* (1.2 × 10^8^ CFU/ml) were used to separately inoculate duplicate vials of 190 µl pooled bovine semen prior to DNA extraction on the KingFisher Flex and screening with the VetMAX rtPCR, as described above. This experiment was performed twice on separate days.

The ability of the VetMAX rtPCR to detect *M. bovis* in the presence of competitive strains in the semen was then evaluated. Two different *M. bovis* strains determined to be genomically divergent (353B08 [1.6 × 10^9^ CFU/ml] and 2B19 [1.4 × 10^9^ CFU/ml]) were serially diluted ten-fold in pooled bovine semen from 10^5^ to 10^2^ CFU/ml, where 20 µl of *M. bovis* was added to 180 µl pooled semen. Another set of ten-fold serial dilutions from 10^5^ to 10^2^ CFU/ml were conducted for each *M. bovis* strain, where 20 µl of *M. bovis* was added to 170 µl pooled semen and each sample was inoculated with 10 µl of 4.6 × 10^8^ CFU/ml *M. bovigenitalium* (129B17). All samples were DNA extracted, without prior centrifugation, using the KingFisher Flex and were screened with the VetMAX rtPCR to determine any impact of *M. bovigenitalium* on *M. bovis* detection sensitivity, compared to the samples containing *M. bovis* alone. This experiment was performed on two separate occasions. PCR-DGGE was also performed in parallel to confirm the presence of both organisms.

In two additional experiments 10^6^ CFU/ml *A. laidlawii* (158B98), *M. bovigenitalium* (NCTC 10122) and *U. diversum* (382B16) were used to separately inoculate pooled semen spiked with 10^7^ CFU/ml *M. bovis* to further assess presence of competitor organisms.

### Statistical analysis

Statistical analysis was performed using STATA 15.0 (Stata Corp. LLC, College Station, Texas, USA). Two-way ANOVA was used to assess the interaction of the DNA extraction methods with centrifugation. Sensitivity, specificity and the respective 95% confidence intervals were calculated using a Diagnostic Test Evaluation Calculator (https://www.medcalc.org/calc/diagnostic_test.php). The Kappa coefficient was calculated using a ‘Quantify Agreement with Kappa’ Calculator (https://www.graphpad.com/quickcalcs/kappa1.cfm). Graphs were generated using GraphPad Prism 8.4.2 (GraphPad Software, Inc., California, USA).

## Results

### Analytical specificity

The DNA from all non-target bacterial species (*n* = 17) consistently returned negative results, with no DNA amplification as indicated by measured FAM signal throughout the 45 cycles of the VetMAX rtPCR (with a cut-off Cq value of 36 for positivity). PCR-DGGE analysis fully confirmed the identification of the mollicutes DNA samples (see Additional file 1). Specificity of the *M. bovis* VetMAX rtPCR was therefore 100% (95% confidence interval [CI] = 80–100%) for this panel.

The VetMAX rtPCR also did not amplify *M. bovis* from DNA extracted from any of the replicate semen samples spiked with *A. laidlawii* [158B98], *M. bovigenitalium* [NCTC 10122] and *U. diversum* [382B16] (see Additional file 2). The specificity of the VetMAX rtPCR in this case was also 100% (95% CI = 74–100%).

### Diagnostic sensitivity and specificity

All 16 samples determined to be positive for *M. bovis* by PCR-DGGE and their replicates, were positive for *M. bovis* DNA when tested in triplicate by the VetMAX rtPCR (Table 1), and by the endpoint *M. bovis*-specific PCR (data not shown). With 16 true positive results and no false negative results (based on PCR-DGGE) the diagnostic sensitivity was 100% (95% CI = 79–100%). However, four samples that consistently tested negative for *M. bovis* by PCR-DGGE, but positive by rtPCR were identified, resulting in a comparative diagnostic specificity of 55.56% (95% CI = 21–86%). One of these four samples (sample #20), which had an average Cq of 25.34, was a lung sample with multiple other mycoplasmas identified by PCR-DGGE and was positive by the endpoint *M. bovis*-specific PCR (Table 1). The remaining three samples positive by the VetMAX rtPCR but negative by PCR-DGGE and the endpoint *M. bovis*-specific PCR had average Cqs between 31.90 and 34.61. The same results were obtained on re-testing. Eight samples were negative for the IPC, where five samples (sample #5, sample #8, sample #10, sample #19 and sample #22) were positive for *M. bovis* with low average Cqs from 11.65 to 13.21. Three semen samples (sample #4, sample #18 and sample #23) were negative for *M. bovis* as well as the IPC, however 1/10 and 1/100 dilutions of the samples did not identify PCR inhibition as there was no amplification of the IPC (data not shown).

Overall, the levels of agreement between the VetMAX rtPCR and PCR-DGGE and between the VetMAX rtPCR and the *M. bovis*-specific PCR were 84.00% (21/25), Kappa coefficient 0.62 (indicating substantial agreement) and 88.00% (22/25), Kappa coefficient 0.69 (indicating moderate agreement), respectively.

**Table 1:** VetMAX^™^ rtPCR results of DNA from UK field samples (n= 25) tested for *M. bovis*

Sample	PCR-DGGE identification	Isolation site	*M. bovis* endpoint PCR	VetMAX™ *M. bovis* rtPCR		Average Cq	SD	SEM
				IPC Cq* (HEX)	*M. bovis* Cq (FAM)			
1	*M. bovis*, *M. dispar*, *M. arginini*, and unidentified bands	Lung	Positive	23.10	16.06	15.96	0.09	0.05
				22.99	15.89			
				23.02	15.94			
2	*M. bovis* and *U. diversum*	Lung	Positive	20.66	21.37	21.51	0.13	0.08
				20.74	21.53			
				20.78	21.63			
3	*M. bovis*, *M. bovigenitalium* and *M. arginini*	Lung	Positive	23.89	18.28	18.33	0.17	0.10
				24.37	18.52			
				23.85	18.20			
4	Unidentified bands	Semen	Negative	No Cq	No Cq	-	-	-
				No Cq	No Cq			
				No Cq	No Cq			
5	*M. bovis*	Lung	Positive	No Cq	10.72	11.65	0.87	0.50
				No Cq	12.44			
				No Cq	11.79			
6	*M. bovis* and *M. dispar*	Lung	Positive	20.65	22.76	22.86	0.09	0.05
				20.73	22.94			
				20.81	22.89			
7	*M. bovis*, *M. bovigenitalium*, *M. bovirhinis* and *M. arginini*	Lung	Positive	33.91	19.49	18.56	0.88	0.51
				36.02	18.45			
				36.14	17.74			
8	*M. bovis*	Lung	Positive	No Cq	12.71	12.78	0.08	0.05
				No Cq	12.87			
				No Cq	12.77			
9	*M. bovis*, *M. dispar*, *M. arginini* and *U. diversum*	Lung	Positive	24.51	22.17	22.13	0.27	0.15
				24.04	21.84			
				24.63	22.37			
10	*M. bovis*	Lung	Positive	No Cq	12.34	12.35	0.19	0.11
				No Cq	12.54			
				No Cq	12.17			
11	*M. bovis*, *M. dispar* and *M. bovirhinis*	Lung	Positive	19.41	34.98	35.50	1.00	0.58
				19.59	36.66			
				19.67	34.87			
12	*U. diversum*	Foetal stomach contents	Negative	30.02	34.79	33.85	0.85	0.49
				30.03	33.15			
				29.97	33.61			
13	*M. bovis*	Cerebrospinal fluid	Positive	21.91	23.96	24.28	0.40	0.23
				22.05	24.15			
				22.55	24.73			
14	*M. bovis* and *M. arginini*	Lung	Positive	19.37	17.48	17.53	0.09	0.05
				19.32	17.47			
				19.47	17.63			
15	No mycoplasma	Semen	Negative	27.29	35.5	34.61	1.04	0.60
				28.99	34.87			
				26.97	33.47			
16	*M. bovis* and *arginini*	Lung	Positive	21.27	17.60	17.51	0.14	0.08
				21.27	17.34			
				21.32	17.58			
17	*M. bovoculi*	Swab	Negative	25.44	36.55	-	-	-
				25.32	No Cq			
				25.38	37.37			
18	Unidentified band	Semen	Negative	No Cq	No Cq	-	-	-
				No Cq	38.15			
				No Cq	No Cq			
19	*M. bovi*s and *M. bovirhinis*	Lung	Positive	No Cq	12.69	12.65	0.14	0.08
				No Cq	12.47			
				No Cq	12.80			
20	*M. bovigenitalium*, *M. dispar* and *U. diversum*	Pooled sample mixed	Positive	20.62	25.18	25.34	0.18	0.11
				20.81	25.31			
				20.90	25.54			
21	*M. bovis* and *M. alkalescens*	Lung	Positive	20.34	16.03	15.93	0.10	0.06
				20.42	15.92			
				20.24	15.83			
22	*M. bovis*, *M. bovigenitalium* and *M. bovirhinis*	Lung	Positive	No Cq	13.15	13.21	0.17	0.10
				No Cq	13.40			
				No Cq	13.08			
23	No mycoplasma	Semen	Negative	No Cq	No Cq	-	-	-
				No Cq	No Cq			
				No Cq	36.95			
24	*M. bovigenitalium*	Lung	Negative	No Cq	No Cq	-	-	-
				38.33	No Cq			
				40.49	No Cq			
25	*M. bovoculi*	Pooled swab	Negative	26.77	32.06	31.90	0.30	0.17
				26.69	32.16			
				26.25	31.48			
PCR positive control	-	-	-	No Cq	23.71	23.71	-	-
PCR negative control	-	-	-	No Cq	No Cq	-	-	-

IPC: internal PCR control; SD: standard deviation, and SEM: standard error of the mean. * The IPC targets bovine DNA

### Analytical sensitivity

**Fig. 1:**
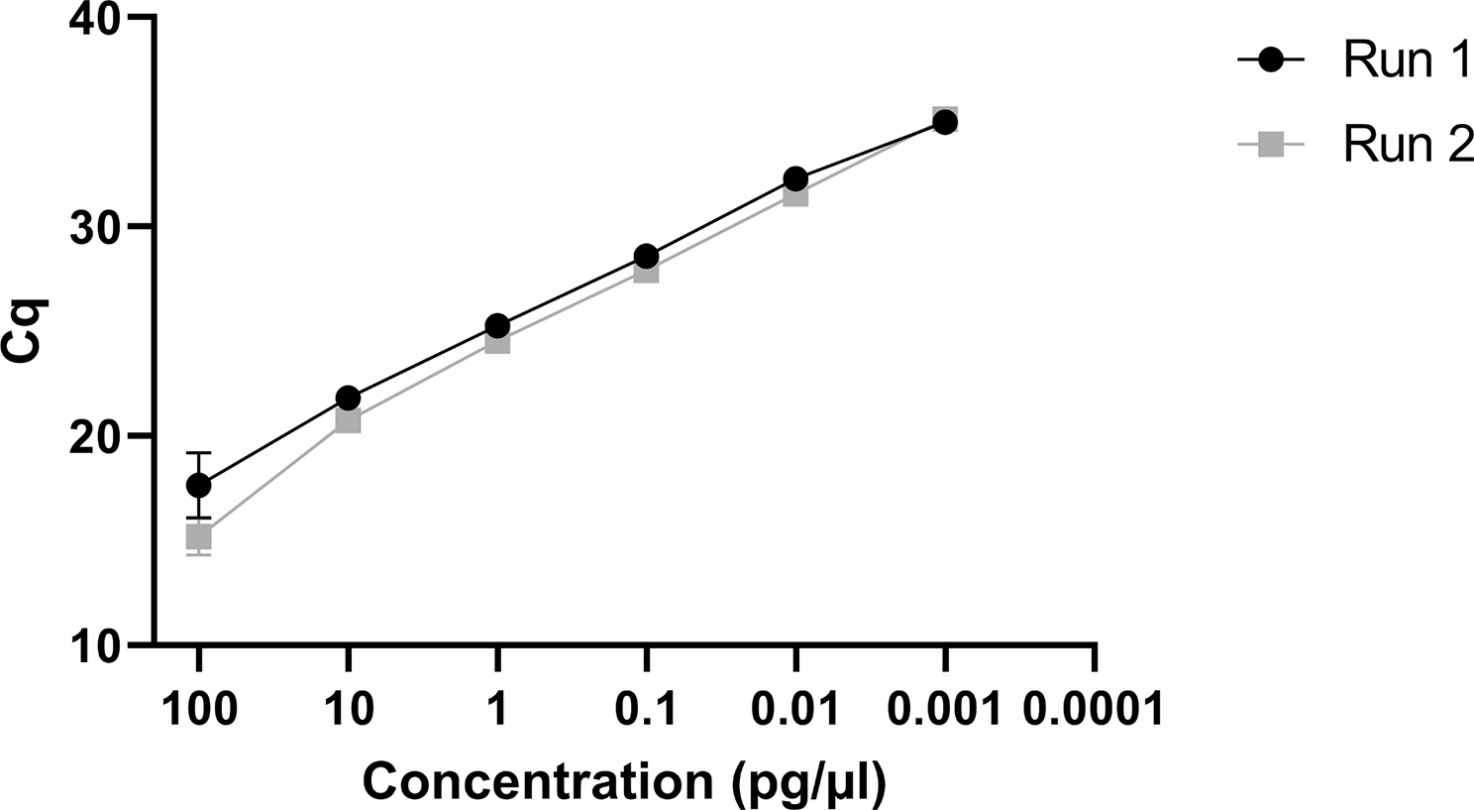
Standard curves of *M. bovis* NCTC 10131 DNA (100-0.0001 pg/µl) detected using the rtPCR. Error bars indicate the standard deviation (based on two independent experiments performed in triplicate)

The limit of detection of the VetMAX™ *M. bovis* rtPCR for *M. bovis* culture as determined by performing ten-fold serial dilutions of purified DNA from *M. bovis* NCTC 10131 (from 100 pg/µl to 0.00001 pg/µl), was 0.001 pg/µl of *M. bovis* DNA (Fig. 1). The limit of detection of PCR-DGGE and the endpoint *M. bovis*-specific PCR was 0.1 pg/µl and 1 pg/µl, respectively (data not shown). Based on the results of the ten-fold dilutions, the PCR efficiency was 87.57%, with a R-squared (R^2^) value of 0.985 and slope of -3.661.

**Fig. 2:**
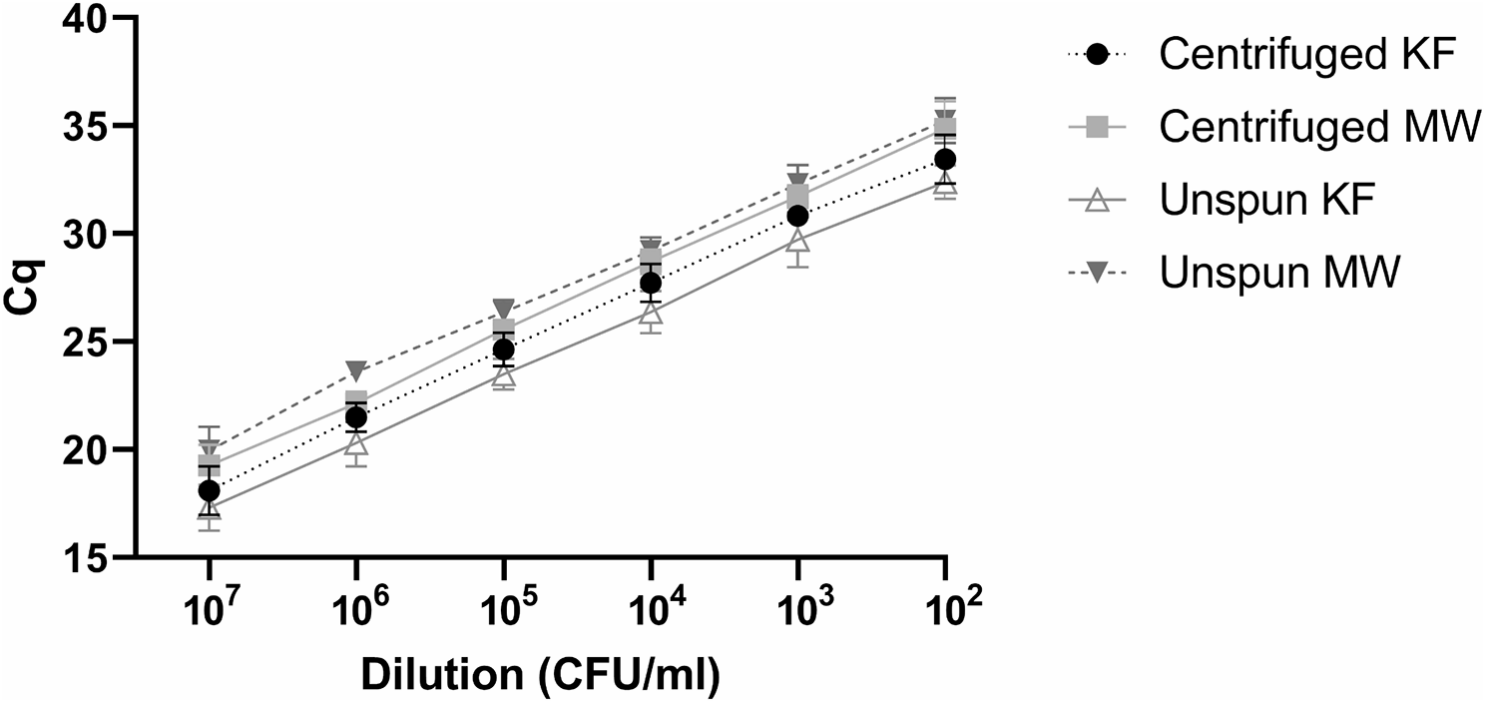
FAM Cq values from centrifuged and unspun semen samples spiked with *M. bovis* (10^2^-10^7^ CFU/ml). KF: KingFisher Flex; MW: Maxwell 16. Error bars indicate the standard deviation (based on three independent experiments performed in duplicate)

*M. bovis* (strain 353B08, representing a commonly occurring clade, A Deeney, unpublished) was serially diluted ten-fold in extended bovine semen straw matrix from 10^7^ CFU/ml to 10^0^ CFU/ml prior to DNA extraction on the KingFisher Flex or Maxwell 16. *M. bovis* was successfully and consistently cultivated in Eaton’s 0134 broth from the 10^7^ to 10^2^ CFU/ml dilutions. *M. bovis* was only inconsistently detectable at the 10^1^ CFU/ml dilution and was absent in the 10^0^ CFU/ml dilution (see Additional file 3). Similarly, the VetMAX rtPCR consistently detected presence of the pathogen from the 10^7^ to 10^2^ CFU/ml dilutions consistently (Table 2; Fig. 2), as well as in the 10^1^ and 10^0^ CFU/ml dilutions but detection was inconsistent at the latter dilutions, therefore the consistent limit of detection was 10^2^ CFU/ml. The limit of detection of the VetMAX rtPCR for detection of DNA extracted from spiked semen using the KingFisher Flex and Maxwell 16, respectively, was lower than that of PCR-DGGE (10^3^ CFU/ml and 10^4^ CFU/ml) and the endpoint *M. bovis*-specific PCR (10^4^ CFU/ml and 10^5^ CFU/ml) (data not shown).

A two-way analysis of variance (ANOVA) indicated that the KingFisher Flex extracted *M. bovis* DNA more efficiently than the Maxwell 16 (*p* < 0.001) based on the lower average Cqs values obtained when centrifuged and unspun samples were compared (Table 3). However, use of a semen centrifugation step to concentrate the sample prior to washing appeared to decrease the Cq values from the Maxwell 16 extraction, while it increased the Cq values from KingFisher Flex (Table 3). Overall, centrifuged samples, where DNA was extracted using the Maxwell 16, had a limit of detection of 5.3 × 10^2^ CFU/ml compared to 7.5 × 10^2^ CFU/ml for unspun samples. For the KingFisher Flex the corresponding limit of detections were 2.2 × 10^3^ CFU/ml and 7.7 × 10^2^ CFU/ml for centrifuged and unspun samples, respectively (Table 2).

**Table 2:** Average Cq and CFU/ml values of *M. bovis*-spiked semen DNA extracted with and without centrifugation

Sample	Average*	Dilution (CFU/ml)					
		10^7^	10^6^	10^5^	10^4^	10^3^	10^2^
Centrifuged KingFisher Flex samples	Cq	18.10	21.48	24.63	27.71	30.82	33.44
	CFU/ml	1.1 × 10^8^	1.5 × 10^7^	1.8 × 10^6^	1.2 × 10^5^	8.5 × 10^3^	2.2 × 10^3^
Centrifuged Maxwell 16 samples	Cq	19.25	22.13	25.55	28.71	31.71	34.86
	CFU/ml	8.7 × 10^7^	9.0 × 10^6^	6.1 × 10^5^	5.1 × 10^4^	5.7 × 10^3^	5.3 × 10^2^
Unspun KingFisher Flex samples	Cq	17.31	20.30	23.49	26.37	29.73	32.38
	CFU/ml	6.7 × 10^7^	7.1 × 10^6^	5.9 × 10^5^	7.0 × 10^4^	6.5 × 10^3^	7.7 × 10^2^
Unspun Maxwell 16 samples	Cq	19.95	23.60	26.36	29.19	32.30	35.25
	CFU/ml	1.4 × 10^8^	6.9 × 10^6^	7.3 × 10^5^	7.8 × 10^4^	7.9 × 10^3^	7.5 × 10^2^

*Based on three independent experiments performed in duplicate

**Table 3:** Average FAM Cq values for each combination of extraction method and centrifugation

Variable(s)	Average Cqs*	95% confidence interval	
KingFisher Flex DNA extraction	25.48	25.29	25.67
Maxwell 16 DNA extraction	27.40	27.21	27.60
No centrifugation	26.35	26.16	26.54
Centrifugation	26.53	26.34	26.73
KingFisher Flex DNA extraction without centrifugation	24.93	24.66	25.21
KingFisher Flex DNA extraction with centrifugation	26.03	25.76	26.30
Maxwell 16 DNA extraction without centrifugation	27.77	27.50	28.04
Maxwell 16 DNA extraction with centrifugation	27.04	26.76	27.31

* These are the average Cq values across the serial dilutions of *M. bovis* spiked in semen

### Detection of *M.bovis* in supernatants from centrifuged samples

*M. bovis* DNA was consistently identified in the supernatants from the higher organism load samples (10^7^ to 10^4^ CFU/ml dilutions) but undetectable at low loads (Table 4). Despite there being detectable *M. bovis* in the supernatant, centrifugation was more efficient for the Maxwell 16 than without centrifugation (*p* < 0.001). However, the KingFisher Flex was the most efficient method overall for extracting *M. bovis* DNA (*p* < 0.001) (Table 3).

**Table 4:** Cqs from centrifuged semen samples spiked with *M. bovis* and their supernatants following DNA extraction

Dilution (CFU/ml)	Run	KingFisher Flex			Maxwell 16		
		Pellet	Supernatant	SD	Pellet	Supernatant	SD
10^7^	Run 1	19.08	22.54	2.45	19.19	24.93	4.06
	Run 2	18.90	25.15	4.42	19.25	26.24	4.94
10^6^	Run 1	21.76	25.48	2.63	21.96	29.78	5.53
	Run 2	22.09	28.20	4.32	22.51	28.38	4.15
10^5^	Run 1	24.96	28.42	2.45	25.71	31.75	4.27
	Run 2	24.99	33.44	5.98	25.77	32.31	4.62
10^4^	Run 1	27.73	32.00	3.02	28.71	34.22	3.90
	Run 2	27.92	35.72	5.52	29.10	34.51	3.83
10^3^	Run 1	31.08	34.51	2.43	32.33	No Cq	-
	Run 2	30.46	No Cq	-	31.43	35.75	3.05
10^2^	Run 1	32.61	No Cq	-	35.56	35.31	0.18
	Run 2	33.95	36.16	1.56	35.03	No Cq	-
10^1^	Run 1	34.75	No Cq	-	No Cq	No Cq	-
	Run 2	No Cq	No Cq	-	No Cq	No Cq	-
10^0^	Run 1	No Cq	No Cq	-	No Cq	No Cq	-
	Run 2	No Cq	No Cq	-	No Cq	No Cq	-

SD: standard deviation

### Specificity of the VetMAX rtPCR: other mollicutes present in semen

**Fig. 3:**
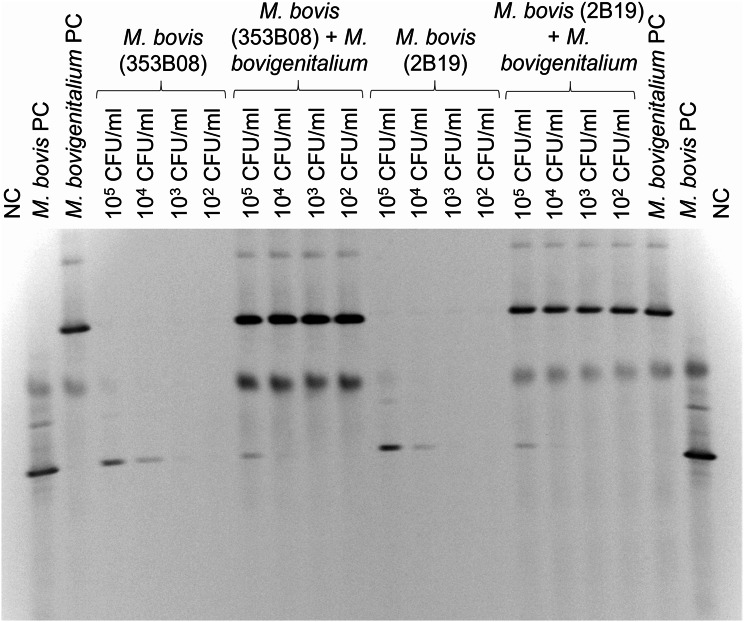
PCR-DGGE of semen samples spiked with *M. bovis* (10^2^-10^5^ CFU/ml) and 10^6^ CFU/ml *M. bovigenitalium*. The positive controls were *M. bovis* NCTC 10131 and *M. bovigenitalium* 10122. The negative control was nuclease-free water. PC: positive control and NC: negative control. The gel was cropped in between lanes 11 and 12 to remove an additional run of *M. bovis* 353B08. The full-length gel is presented in Supplementary Fig. 1

When spiked in semen in a range of ten-fold dilutions from 10^5^ CFU/ml to 10^2^ CFU/ml in the presence of a moderate concentration of *M. bovigenitalium* (strain 129B17; 10^6^ CFU/ml), the sensitivity of detection of the VetMAX rtPCR for *M. bovis* was not adversely affected by the presence of the other organism (Table 5; Fig. 3; SD 0.01 to 0.96). However, the ability of PCR-DGGE to detect *M. bovis* in the presence of *M. bovigenitalium* was reduced, due to competition for the mycoplasma specific 16S rRNA gene-based primers (Fig. 3).

**Table 5:** Effect of *M. bovigenitalium* as a competitor in semen on the *M. bovis* rtPCR efficiency

Sample	Run	Dilution (CFU/ml)				
		10^5^	10^4^	10^3^	10^2^	
*M. bovis* (353B08)	**Run 1**	25.75	28.00	31.38	34.37	
*M. bovis* (353B08) + *M. bovigenitalium* (129B17)		25.45	28.07	31.43	33.70	
SD		0.21	0.05	0.03	0.47	
*M. bovis* (353B08)	**Run 2**	23.66	26.55	29.57	32.22	
*M. bovis* (353B08) + *M. bovigenitalium* (129B17)		23.48	26.84	29.66	32.24	
SD		0.13	0.21	0.06	0.01	
*M. bovis* (2B19)	**Run 1**	24.32	27.29	29.97	33.05	
*M. bovis* (2B19) + *M. bovigenitalium* (129B17)		24.26	27.25	29.83	32.44	
SD		0.04	0.03	0.10	0.43	
*M. bovis* (2B19)	**Run 2**	24.12	27.40	30.17	33.34	
*M. bovis* (2B19) + *M. bovigenitalium* (129B17)		24.15	27.48	29.83	31.98	
SD		0.02	0.06	0.24	0.96	

SD: standard deviation

Real-time PCR detection of *M. bovis* was also unaffected by the presence of *M. bovigenitalium* (NCTC 10122) as well as other mollicute strains *A. laidlawii* (strain 158B98), and *U. diversum* (strain 382B16), spiked in semen at 10^6^ CFU/ml together with *M. bovis* (353B08) (see Additional file 5).

## Discussion

For the purposes of screening for specific pathogens rtPCRs have rapidly become the method of choice owing to faster turnaround time and enhanced sensitivity offered over traditional endpoint PCRs. The present study first assessed that the VetMAX™ *M. bovis* PCR was suitably specific as one of three rtPCRs approved by MPI for detection of *M. bovis*, where two DNA extraction methods were compared (Maxwell 16 [Promega] and KingFisher Flex [ThermoFisher Scientific]) using semen that was either centrifuged or unspun prior to DNA extraction.

The VetMAX™ *M. bovis* rtPCR had 100% sensitivity and was found to have a limit of detection of 0.001 pg/µl *M. bovis* DNA from pure culture and 10^2^ CFU/ml *M. bovis* in semen, ranging from 5.3 × 10^2^ CFU/ml to 2.2 × 10^3^ CFU/ml for centrifuged samples processed on the Maxwell 16 and KingFisher Flex, respectively, and 7.5 × 10^2^ CFU/ml to 7.7 × 10^2^ CFU/ml for unspun samples, respectively. The limit of detection of the VetMAX™ *M. bovis* rtPCR was similar to the 2.2 × 10^2^ CFU/ml recently reported by researchers in New Zealand [19] and the 1.0 × 10^2^ CFU/ml to 1.0 × 10^3^ CFU/ml reported by three laboratories participating in a European interlaboratory comparison of *M. bovis* PCR assays [22]. To our knowledge, the VetMAX™ *M. bovis* rtPCR appears to have a superior limit of detection to other rtPCRs previously performed for analysis of bovine semen, including a multiplex rtPCR designed by Parker et al. [32]. targeting the *uvrC* gene of *M. bovis* (as well as the *rpoB* gene of *M. californicum* and the 16–23 S rRNA intergenic spacer region of *M. bovigenitalium*), with a reported detection limit of 1.3 × 10^5^ CFU/ml and 1.3 × 10^7^ CFU/ml for *M. bovis* in semen when alone and in the presence of *M. californicum* and *M. bovigenitalium*, respectively. A dual-target TaqMan rtPCR system of McDonald (2012) detected *M. bovis* in semen by targeting the housekeeping genes *fusA* and *oppD/F*, reported a detection limit of 3.1 × 10^3^ CFU/ml [34].

The testing of a panel of 25 DNA samples from different field sample types submitted for diagnostic testing identified eight samples that were negative for the IPC, which targets bovine DNA. Five of these samples had very high levels of *M. bovis* DNA (average Cqs 11.65 to 13.21), which may have caused false negative IPC results due to amplification bias as the *M. bovis* DNA had a higher probability of being amplified [40]. The remaining three samples were negative for *M. bovis* and subsequent dilutions appeared to rule out PCR inhibition due to no amplification of *M. bovis* nor the IPC. One possibility may be DNA degradation due to these samples being in long-term storage at -20 °C for several years as a study by Baoutina et al. (2019) showed DNA stored in Tris-EDTA buffer at low levels (~ 20 pg/ml) exhibited substantial degradation within weeks of storage at -20 °C [41], Alternatively, freeze-thawing of the samples over time may have led to DNA degradation as a study by Yoo et al. (2011) showed that lambda DNA in Tris-EDTA buffer degraded by 10% following one freeze-thaw and had degraded by 75% following 20 freeze-thaws [42]. Therefore, if these semen samples had low levels of bovine DNA, it is likely degradation from long term storage prevented detection by the VetMAX rtPCR.

In this study *M. bovis* DNA extracted using the KingFisher Flex from non-centrifuged samples was most efficiently detected by the VetMAX rtPCR. In contrast, use of a centrifugation step improved extraction efficiency of *M. bovis* DNA on the Maxwell 16 assessed by FAM Cq values following the VetMAX rtPCR. Both protocols included a heat-lysis step in the presence of proteinase K prior to DNA extraction, although they differed in the centrifugation speeds. The differences in efficiency of *M. bovis* detection observed for centrifuged semen may be attributed to *M. bovis* cells being lost using the recommended KingFisher Flex protocol 3,000 g centrifugation step, compared with our in-house standard 17,000 g when using the Maxwell 16. The published New Zealand IHS requirement for minimally processed uncentrifuged semen straw samples [36], did not adversely affect the ability of the test to detect *M. bovis* at low levels in semen, particularly using the KingFisher with the MagMax core kit. However, while this stipulation does not appear to confer a substantial advantage regarding test sensitivity, it minimises sample handling and processing of this pathogen, which is handled in high containment in New Zealand due to its unwanted status.

Additionally, the requirement for unwashed and uncentrifuged semen could potentially pose a risk of PCR inhibitors remaining in the semen samples as semen contains very high levels of citric acids, DNA, fructose protein and potassium ions, which could reduce PCR efficiency resulting in higher Cq values and limits of detection [32, 33]. However, we found no evidence for PCR inhibition (assume no failures for internal controls), although both the Maxwell 16 and Kingfisher Flex protocols employed a heat-lysis step prior to automated DNA extraction as a precaution. Jaramillo and colleagues observed that PCR inhibition mainly impacted crude DNA preparation methods, such as the InstaGene (BioRad) protocol, whereas DNA extracted using the KingFisher MagMax Core protocol was unaffected by PCR inhibition [19], which was consistent with our findings. Furthermore, our automated DNA extraction procedures have been used for a variety of tissue and sample types, which have previously included semen and milk, with no major PCR inhibition detected. Therefore, the lack of PCR inhibitors in our study suggests the automated nucleic acid extraction methods used were effective in purifying the DNA and removing PCR inhibitors.

Investigation of test sensitivity was limited by challenges associated with obtaining semen samples naturally infected with *M. bovis* for our study. Instead, the testing was conducted with semen released from processed straws that were pooled, spiked and mixed thoroughly prior to testing. To compare analytical specificity afforded by the VetMAX rtPCR under test, historical DNA prepared using our standard approach [22] from a variety of clinical sample types, with known PCR-DGGE profiles were tested using the VetMAX rtPCR, with 100% analytical specificity for the panel tested, where DNA from other Mollicutes (including *M. bovigenitalium* and *U. diversum*) and non-Mollicute species were not amplified. However, the VetMAX rtPCR detected *M. bovis* DNA in five field samples where PCR-DGGE did not identify this pathogen, particularly those harbouring different *Mycoplasma* spp. High Cqs (> 25) were observed in these samples negative by PCR-DGGE but positive by the VetMAX rtPCR. This finding is attributable to a combination of differences in sensitivity (0.001 pg/µl DNA compared to 0.1 pg/µl DNA for PCR-DGGE) between real-time and endpoint PCRs (data not shown), but also as PCR-DGGE targets the V3 region of 16S rRNA [21], *M. bovis* may be outcompeted if present in low levels in a sample containing other mollicutes with higher abundance. As the VetMAX rtPCR under test targets the *M. bovis*-specific *polC* gene no competition with other species for the PCR reagents is expected, allowing *M. bovis* to be detectable at relatively low levels even in the presence of other mollicutes. Moreover, for PCR DGGE only 1 µl DNA template is used per 50 µl PCR reaction for PCR-DGGE, compared to an unusually high 5 µl in 25 µl for the VetMAX rtPCR further impacting sensitivity. However, the PCR-DGGE test used at APHA simultaneously identifies multiple *Mycoplasma* and related species from several sample and tissue types, irrespective of successful culture and has proven invaluable for mycoplasma diagnostics in a variety of host animal species, including the ability to detect *M. bovigenitalium* and *Ureaplasma diversum* which can be found in semen and are also associated with bovine infertility, but may also be identified in cases of bovine respiratory disease [21]. Despite the potential for false negatives in mixed samples due to competition for PCR reagents, it would be beneficial to run PCR-DGGE in parallel to the VetMAX rtPCR due to its ability to identify other mycoplasmas present in the semen.

The detection of *M. bovis* did not appear to be affected by competition with 4.6 × 10^6^ CFU/ml *M. bovigenitalium* as *M. bovis* was still detectable from 1.5 ± 0.1 × 10^5^ CFU/ml to 1.5 ± 0.1 × 10^2^ CFU/ml, with insignificant differences in the Cq values when compared to samples with *M. bovis* alone. This pathogen was chosen as a competitor due to it being the most common mycoplasmal cause of infertility in cattle [12, 43, 44] and its isolation from semen [45, 46]. Furthermore, 10^6^ CFU/ml of representative strains of *A. laidlawii* (4.3 × 10^8^ CFU/ml) and *U. diversum* (1.2 × 10^8^ CFU/ml), both organisms that have also been isolated from semen [45–47], had no deleterious effect on the ability of the assay to identify that 10^2^ to 10^5^ CFU/ml *M. bovis* were present in the sample. A study by Becker et al. (2020) [48] successfully amplified *M. bovis* in double-nasal swabs from veal calves (51.30%, 59/115) from 21 feedlots where bovine respiratory disease occurred using the VetMAX™ *M. bovis* PCR kit, where in 13 instances Pasteurellaceae were co-isolated, suggesting *M. bovis* was frequently detected in samples where other pathogens are highly abundant. Moreover, a study by Djebala et al. 2020 [49] identified *M. bovis* (8.33%, 6/72) in combination with Bovine Herpesvirus 4 in peritoneal exudate from cows affected by parietal fibrinous peritonitis, when using the VetMAX™ *M. bovis* PCR kit. Together, these studies demonstrate that the *M. bovis polC* DNA target is unlikely to be outcompeted during PCR amplification using this test in different clinical sample types from cattle.

Limitations to this study include the inability to test raw semen as well as semen naturally infected with *M. bovis*. Additionally, freezing-thawing semen straws after spiking with *M. bovis* to mimic the real practical conditions was not possible as we did not have the facilities to prepare, and nitrogen freeze such straws. Application of both DNA extraction and rtPCR testing procedures on raw samples; replication of this study using naturally infected semen to make the experiments more realistic as well as consideration of the impact of freezing-thawing semen after spiking with *M. bovis* to more closely align standard preparation procedures are warranted in future studies. Furthermore, as the KingFisher DNA extraction protocol was modified by experts’ recommendations, this may have skewed the results. However, this modified protocol has now been published by ThermoFisher to be used for raw semen [50] so their protocol for semen straws should be tested in a future study. Finally, as two semi-automated DNA extraction systems were compared in this study, the results of this study may not be relevant to smaller or less equipped laboratories. Therefore, the use of the manual Qiagen DNA-Mini Kit, which was also suggested by MPI for DNA extraction, should be compared in a future study to reveal if lower-cost options can detect *M. bovis* in semen samples with equal sensitivity and specificity.

## Conclusions

In conclusion, our findings demonstrated the efficient detection of *M. bovis* in artificially infected bovine semen straws by the VetMAX™ *M. bovis* rtPCR kit under conditions that comply with NZ’s IHS. The VetMAX rtPCR offers a consistent limit of detection of 10^2^ CFU/ml as well as a rapid turnaround, with results obtainable after around 2 hours, compared to 2–3 days for PCR-DGGE and 4 h for conventional PCR and is less labour intensive. The VetMAX rtPCR also offers a cheaper alternative to PCR-DGGE, due to the latter having additional costs for bacterial culture, preparation of gels and staff time, and it requires less time than conventional PCR due to lack of manual analysis. However, unlike PCR-DGGE it can only detect *M. bovis* and no other *Mycoplasma* spp. so other important urogenital mollicutes associated with reproductive disease in cattle and found in semen would be missed if only using this test. These other mollicutes are worthy of future consideration through development of syndromic multiplex tests Although the VetMAX rtPCR can be used to test DNA extracted from both the Maxwell 16 and KingFisher Flex, the latter platform appeared to extract DNA with slightly more efficiently than the Maxwell 16 (*p* < 0.001) and DNA extraction was marginally improved by not centrifuging the samples, whereas centrifugation had the opposite effect for the Maxwell 16 (*p* < 0.001). Therefore, the IHS’ requirement for the use of the KingFisher Flex for DNA extraction of bovine semen straws without centrifugation is not unfounded. Nevertheless, DNA extraction and PCR protocols outlined in the study have been validated in our laboratory as fit for purpose for screening for *M. bovis* in extended bovine semen destined for export to New Zealand.

## Electronic supplementary material

Below is the link to the electronic supplementary material.

Supplementary Material 1: Title: VetMAX^™^ real-time PCR results of a specificity panel of DNA samples containing non-*M. bovis* species. Description of data: Additional file 1 of VetMAX^™^*M. bovis* real-time PCR results of a specificity panel of DNA samples containing non-*M. bovis* species (*n* = 17), which were tested in triplicate in two separate runs.

Supplementary Material 2: Title: VetMAX™ real-time PCR results of specificity panel of semen samples spiked with non-*M. bovis* species. Description of data: Additional file 2 of VetMAX™ *M. bovis* real-time PCR results of a specificity panel of semen samples spiked with *A. laidlawii*,* M. bovigenitalium and U. diversum*, tested in two separate runs.

Supplementary Material 3: Title: Growth of *M. bovis* (10^7^ to 10^0^ CFU/ml) spiked in semen in Eaton’s 0134 broth. Description of data: Additional file 3 of growth results of *M. bovis* (10^7^ to 10^0^ CFU/ml) spiked in semen in Eaton’s 0134 broth prior to DNA extraction.

Supplementary Material 4: Title: Amount of *M. bovis* (CFU/ml) present in the supernatant following centrifugation during DNA extraction. Description of data: Additional file 4 showing the amount of *M. bovis* (CFU/ml) present in the supernatant following centrifugation compared to the centrifuged samples for each DNA extraction method.

Supplementary Material 5: Title: Comparison of Cqs from *M. bovis* spiked into semen with or without non-*M. bovis* strains. Description of data: Additional file 5 showing the comparison of Cqs from *M. bovis* spiked into semen with or without non-*M. bovis* strains (*A. laidlawii*,* M. bovigenitalium and U. diversum*).

Supplementary Material 6: Title: Fig. 3: PCR-DGGE of semen samples spiked with *M. bovis* (10^2^-10^5^ CFU/ml) and 10^6^ CFU/ml *M. bovigenitalium*. The positive controls were *M. bovis* NCTC 10131 and *M. bovigenitalium* 10122. The negative control was nuclease-free water. PC = positive control and NC = negative control. Description: Full-length gel of Fig. 3, where the area cropped from the gel containing a second run of *M. bovis* 353B08 is outlined in black. The area outside of the grey outline was also cropped.

## Data Availability

The full datasets used and/or analysed during the current study are available from the corresponding author on reasonable request.

## Abbreviations

APHA: Animal and Plant Health Agency
Defra: Department for Environment, Food and Rural Affairs
DGGE: Denaturing gradient gel electrophoresis
IHS: Import Health Standard
MPI: Ministry of Primary Industries
