# Supplementary figures and images for: Comparison of DNA extraction procedures for detection of Mycoplasma bovis directly from extended bovine semen straw samples using a commercial M. bovis PCR
Source: BMC Vet Res. 2024 Oct 26;20:491. doi: 10.1186/s12917-024-04333-z (PMC11515183; doi:10.1186/s12917-024-04333-z)

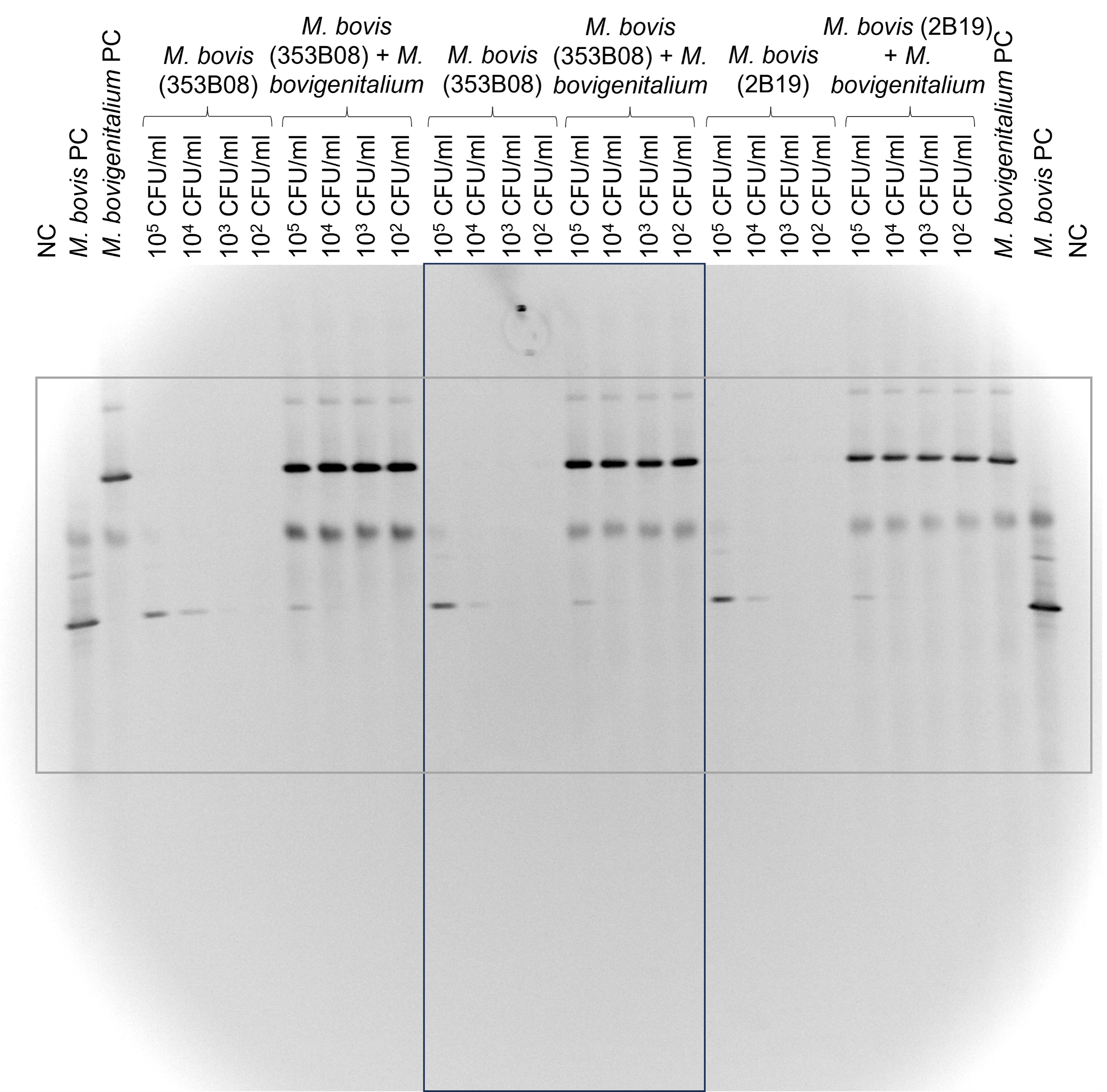

Supplement: Supplementary file 6 — Supplementary Material 6: Title: Fig. 3: PCR-DGGE of semen samples spiked with M. bovis (102-105 CFU/ml) and 106 CFU/ml M. bovigenitalium. The positive controls were M. bovis NCTC 10131 and M. bovigenitalium 10122. The negative control was nuclease-free water. PC = positive control and NC = negative control. Description: Full-length gel of Fig. 3, where the area cropped from the gel containing a second run of M. bovis 353B08 is outlined in black. The area outside of the grey outline was also cropped. [file 12917_2024_4333_MOESM6_ESM.png]
